# 基于精氨酸酶切的蛋白质C端肽段富集方法的优化及评估

**DOI:** 10.3724/SP.J.1123.2021.03030

**Published:** 2022-01-08

**Authors:** Xiaoxiao ZHAO, Hao HU, Wensi ZHAO, Ping LIU, Minjia TAN

**Affiliations:** 1.南京中医药大学新中药学院, 江苏 南京 210023; 1. School of Chinese Materia Medica, Nanjing University of Chinese Medicine, Nanjing 210023, China; 2.中国科学院上海药物研究所新药研究国家重点实验室, 上海 201203; 2. State Key Laboratory of Drug Research, Shanghai Institute of Materia Medica, Chinese Academy of Sciences, Shanghai 201203, China; 3.中国科学院大学, 北京 100049; 3. University of Chinese Academy of Sciences, Beijing 100049, China

**Keywords:** 化学乙酰化, 蛋白质C端组学, 反向富集, 固相萃取枪头膜片过滤柱, 生物质谱, chemical acetylation, C-terminomics, negative enrichment, StageTip cartridge, biological mass spectrometry

## Abstract

基于聚合物的蛋白质C端反向富集策略是用于研究蛋白质C端最为广泛的策略之一。目前,基于胰蛋白酶(trypsin)切割精氨酸残基C端(ArgC型酶切)的蛋白C端组学方法对蛋白质C端的鉴定深度仍有待提高。为解决这一问题,该研究对此方法进行了优化和评估:建立了基于“V型”过滤装置的“一锅法”富集流程,避免了副反应的干扰,缩短了样本的制备时间;优化了蛋白水平乙酰化反应条件,最大限度地降低了丝氨酸、苏氨酸、酪氨酸残基上的副反应,提高了肽段鉴定的可信性;优化了基于固相萃取枪头膜片过滤柱(StageTip柱)的样品分离过程,使C端肽段的鉴定深度增加至原来的4倍。通过以上优化,按照肽段水平错误发现率(FDR)<0.01、离子分数(ion score)≥20,且C端带有乙醇胺修饰的数据筛选标准,从人HEK 293T细胞中共鉴定出696个蛋白质C端。若仅要求肽段水平FDR<0.01,鉴定数目进一步增加到933个,这是基于聚合物富集策略的蛋白质C端组学方法所得的最大数据集之一。探索了胰蛋白酶镜像酶(LysargiNase)切割精氨酸残基N端(ArgN型酶切)与不同肽段N端衍生化修饰组合对蛋白质C端鉴定数目和种类的影响,“LysargiNase酶切+肽段N端乙酰化”新策略在原有“胰蛋白酶酶切+肽段N端二甲基化”策略的基础上将鉴定蛋白质C端的种类提升了47%。综上,该研究通过对基于Arg型酶切的蛋白C端组学方法的优化,提升了C端肽段的鉴定深度,扩大了C端肽段鉴定的覆盖范围。该方法将有望成为系统性表征蛋白质C端的有力工具。

## Abstract (en)

As unique biomarkers, protein C-termini are involved in various biological processes such as protein trafficking, subcellular relocation, and signal transduction. Dysregulation of protein C-terminal status is critical during the development of various diseases, including cardiovascular, neurodegenerative, and metabolic diseases and cancer. Thus, global profiling of protein C-termini is of great value in providing mechanistic insight into biological or pathological processes, as well as for identifying potential new targets for therapeutic treatment.

Polymer-based negative enrichment is a prominent C-terminomics strategy with advantages of universal applicability and parallel sample preparation. Compared with other methods of such a strategy, the profiling depth of the approaches based on enzymatic cleavage of Arg residues still needs to be improved. This greatly limits our understanding of the physiological functions and molecular mechanisms of C-termini. To add a more powerful tool for C-terminomics, Arg cleavage-based negative enrichment C-terminomics was optimized and evaluated.

First, the sample preparation process was optimized. A one-pot enrichment platform based on a V-shaped filter was established, which reduced sample loss, avoided cross-contamination between reactions, and shortened sample preparation time. In addition, the protein-level acetylation conditions were investigated with the optimal labeling conditions as follows: triple coupling using 5 mmol/L Ac-NHS at pH 7.0 and 500 mmol/L ammonium for 15 min provided minimized acetylation rates (acetylation labeling efficiencies of Ser, Thr, and Tyr were lower than 4%, 2%, and 1%, respectively), along with the highest peptide-spectrum match number and satisfactory Lys labeling efficiency (up to 98%). These optimized conditions would not only minimize acetylation, but also facilitate the identification of C-terminal peptides.

Second, it was speculated that the unexpected low identification rate was primarily caused by the interference of the large number of organic compounds accumulated during the peptide-level reactions, including reagents, organic buffering agents, and their complex side-reaction products. Therefore, the conditions for StageTip-based fractionation, including pH, the amount of Empore C18 beads, and the number of fractions, were optimized. As a result, by separating the sample enriched from 300 μg proteome into seven fractions, sample complexity was largely decreased and a total of 696 C-termini were identified in duplicates from strict data filtration, that is, percolator false discovery rate (FDR)<0.01, ion score≥20, and C-terminal amidation by ethanolamine. If only peptide FDR<0.01 was considered, the identified C-termini further increased to 933, which was among the largest C-terminome datasets obtained from the polymer-based strategy. Furthermore, compared with the results of a previous study, the optimized method would be a practical strategy for broader C-terminome coverage.

Finally, to further broaden the coverage of the sub-C-terminome generated by Arg-specific cleavage, this study explored a new method in which ArgN-specific cleavage (cleavage at the N-terminal of Arg by LysargiNase) was combined with different N-terminal protections (dimethylation and acetylation). Among all the combinations, the additional use of the “LysargiNase+N-terminal acetylation” method increased 47% more identifications of unique C-termini on the basis of “trypsin+N-terminal demethylation” and the two covered 87% of the total C-termini. Therefore, the parallel use of the two methods would further expand the coverage of Arg-cleaved C-terminal peptides. With the analysis of the physicochemical properties of the peptides identified by the two methods, the reason why the C-terminal peptides identified by different strategies are complementary was explained.

In conclusion, in this study, the optimized C-terminomics platform can deeply profile Arg cleavage-generated C-terminal peptides using a polymer-based approach. This method provides a powerful tool for the global characterization of protein C-termini.

蛋白质C端及其翻译后修饰(PTMs,如脂质化、乙酰化、磷酸化等)参与多种生物学过程,如蛋白质定位、蛋白质相互作用、维持蛋白质稳定性等^[1,2,3,4]^。蛋白质C端异常会引起代谢性疾病、神经退行性疾病、心血管疾病等^[5,6,7,8]^多种疾病。因此,蛋白质C端的系统性表征对于生物学机制的解析具有重要意义。

尽管“鸟枪法”蛋白质组学在全蛋白质组分析方面有很大优势,但由于C端肽段占比少、离子化效率低,蛋白质C端的分析效率仍比较低。近年来,基于“鸟枪法”蛋白质组学发展形成的“蛋白质C端组学(C-terminomics)”技术使系统表征蛋白质C端成为可能。该技术主要有两种策略:1)通过对蛋白质C端羧基进行选择性标记并进行亲和富集,直接捕获蛋白质C端的正向富集策略^[9,10,11]^; 2)通过化学衍生化和多种分离技术去除蛋白N端和内部肽段,进一步洗脱获得蛋白质C端的反向富集策略^[12,13]^。后者不仅可以富集普通C端肽段,还可同时富集蛋白质C端*α*-COOH上含有PTMs的C端肽段。尤其是利用基于聚合物的反向富集策略^[14]^进行蛋白质C端的研究,可以实现多个样本平行操作,大大提高了样本的制备通量,并且不需专门的仪器设备,在普通生化实验室即可完成,应用范围更加广泛。

近年来,研究人员基于聚合物富集策略,发展了多种相关方法,通过不断优化,改善了C端肽段的鉴定深度^[15,16,17,18,19,20]^。Zhang等^[18]^通过胰蛋白酶(trypsin)切割精氨酸残基C端(ArgC型酶切)的方式,获得了369个蛋白质C端;Du等^[16]^通过重组赖氨酰肽链内切酶(LysC)切割赖氨酸残基C端(LysC型酶切)的方式,获得了781个蛋白质C端;在本课题组前期LAACTer策略研究^[15]^中,利用胰蛋白酶镜像酶(LysargiNase)切割赖氨酸和精氨酸残基N端(LysN/ArgN型酶切),获得了834个蛋白质C端。这些方法使用不同的特异性酶切方式,因此理论上对C端肽段有着各自独特的鉴定范围,具有一定的互补性。而相比之下,基于精氨酸切割(Arg型酶切)的方法对C端肽段的鉴定深度仍有待提高。随着蛋白质C端在疾病中的作用越来越受到关注,为了鉴定一些重要的蛋白质C端,基于Arg型酶切的蛋白质C端组学方法在鉴定深度上亟待进一步突破。

为解决这一问题,本研究对基于Arg型酶切方法进行了优化和评估。建立了基于“V型”过滤装置的“一锅法”富集平台,并对蛋白水平乙酰化的反应条件进行了系统性的优化。提出了过量有机小分子可能是聚合物反向富集策略中干扰C端肽段鉴定的关键因素,针对性地优化了基于固相萃取枪头膜片过滤柱(StageTip)的样品分离过程,并和已报道的研究结果进行了对比和评估;探索了使用不同酶切方式与不同肽段衍生化修饰的组合对蛋白质C端鉴定数目和种类的影响,旨在利用“LysargiNase酶切+肽段N端乙酰化”策略进一步扩大蛋白质C端的鉴定范围。

## 1 实验部分

### 1.1 仪器、试剂与材料

JY92-IIN细胞粉碎机购于宁波新芝科技股份有限公司;5424R高速离心机购于德国Eppendorf AG公司;SPD111V-230真空离心浓缩仪、Q-Exactive质谱、Orbitrap Fusion质谱、纳升液相色谱EASY-nLC 1000购于美国Thermo Fisher Scientific公司。

蛋白酶抑制剂购于瑞士Roche公司;*N*-羟基琥珀酰亚胺乙酸酯(Ac-NHS)和乙醇胺(EA)购于北京百灵威科技有限公司;LysargiNase和胰蛋白酶购于北京华利世科技有限公司;氯化钙(CaCl_2_)、盐酸胍(GnHCl)、*N*-羟基琥珀酰亚胺(NHS)和*N*-(3-(二甲基氨基)丙基)-乙基二亚胺盐酸(EDC)购于国药集团化学试剂有限公司;4-(2-羟乙基)哌嗪-1-乙磺酸(HEPES)、2-(*N*-吗啉代)乙磺酸(MES)、氰基硼氢化钠(NaBH_3_CN)、二硫苏糖醇(DTT)、碘乙酰胺(IAA)、三氟乙酸(TFA)、聚丙烯基胺(PAA)、甲醛(HCHO)、甲酸铵(AF)均购于美国Sigma公司。Durashell C18填料购于天津博纳艾杰尔科技有限公司;Empore C18固相萃取膜片购于美国3M公司;Amicon Ultra-0.5 filters(10 kDa 截止)超滤管和ZipTip C18脱盐柱购于美国Millipore公司。

### 1.2 蛋白质提取与还原烷基化

将人HEK 293T细胞培养于含有10%胎牛血清、100 U/mL青霉素和100 mg/L链霉素双抗的DMEM培养基中。向获得的湿细胞沉淀中加入10倍体积裂解液(3 mol/L GnHCl、100 mmol/L HEPES、2%(质量分数)蛋白酶抑制剂,pH 7.0),冰上静置30 min后使用细胞破碎仪进行超声处理,以15000 r/min离心10 min,获取上清。按照BCA方法进行蛋白质浓度定量。加入终浓度为5 mmol/L的DTT,于56 ℃反应30 min,最后加入终浓度为15 mmol/L的IAA,室温避光反应30 min。

### 1.3 蛋白水平乙酰化

取100 μg还原烷基化后的样本,用3 mol/L GnHCl和100 mmol/L HEPES,pH 7.0的缓冲液将样本稀释至1 μg/μL。然后加入终浓度为5 mmol/L的Ac-NHS(pH 7.0),室温反应30 min,重复反应3次。再加入终浓度为500 mmol/L的氨水(NH_3_·H_2_O),室温反应15 min。按蛋白质与酶的质量比为50∶1添加胰蛋白酶,调pH 8.0, 于37 ℃孵育16 h。样品转干后,取10 μg样本用ZipTip C18脱盐柱进行除盐。每组样本进行2次技术重复。

### 1.4 蛋白水平酰胺化

取300 μg还原烷基化后的样本,置于0.5 mL超滤管中,加入缓冲液A(100 mmol/L HEPES、3 mol/L GnHCl, pH 7.0)至500 μL,在11600 r/min的转速下超滤至50 μL,重复两次。使用缓冲液A调节样本质量浓度至1 μg/μL,加入终浓度为5 mmol/L的Ac-NHS,室温反应30 min,重复反应3次。再加入缓冲液B(1 mol/L EA、2 mol/L GnHCl、0.2 mol/L MES, pH 6.0)至500 μL,在11600 r/min的转速下超滤至50 μL。使用缓冲液B调节样本质量浓度至1 μg/μL,加入终浓度为20 mmol/L的NHS和100 mmol/L的EDC,于37 ℃反应2 h。再次补加相同终浓度的EDC,反应2 h。

### 1.5 酶切消化

在上述样本中加入胰蛋白酶酶切体系(20 mmol/L HEPES、0.4 mol/L GnHCl, pH 8.0)至500 μL,在11600 r/min的转速下超滤至50 μL,重复3次。使用酶切体系调节样本质量浓度至1 μg/μL,按蛋白质与酶的质量比为50∶1添加胰蛋白酶,于37 ℃孵育16 h。再按蛋白质与酶的质量比为100∶1添加胰蛋白酶,于37 ℃孵育4 h。

在研究不同酶切方式和不同肽段衍生化修饰的组合对C端肽段鉴定数目和种类影响的实验中,引入LysargiNase代替胰蛋白酶进行酶切消化,其酶切体系为20 mmol/L HEPES和10 mmol/L CaCl_2_, pH 8.0,其他条件与胰蛋白酶酶切处理方式相同。

### 1.6 肽段水平衍生化修饰与蛋白质C端富集

对胰蛋白酶酶切消化产生的肽段分别进行乙酰化和二甲基化反应。LysargiNase酶切后的样本进行同样的修饰反应。对于乙酰化修饰,加入终浓度为5 mmol/L的Ac-NHS, pH 7.0,室温反应30 min,重复反应3次;对于二甲基化修饰,加入终浓度为20 mmol/L的HCHO和10 mmol/L的NaBH_3_CN,调节pH 6.0,于37 ℃反应2 h,再次加入相同浓度的HCHO和NaBH_3_CN,反应2 h。每组样本进行2次技术重复。

将反应完毕后的样本在SPD111V-230真空离心浓缩仪中浓缩至150 μL,各自加入150 μL 2 mol/L PAA、50 μL乙腈以及终浓度为20 mmol/L的NHS和100 mmol/L的EDC,于37 ℃反应2 h,补加相同终浓度的EDC,反应2 h。在11600 r/min的转速下离心收集滤液,加入15%(v/v)乙腈水溶液至300 μL,相同转速下离心收集滤液,重复两次。将滤液合并转干冻存。

### 1.7 基于StageTip的样品分离

配制碱性溶液体系:溶液A为98%(v/v)乙腈水溶液(含5 mmol/L AF),溶液B为5 mmol/L AF水溶液(pH 10.0)。

碱性StageTip柱的制备:将Empore C18固相萃取膜片置于200 μL枪头底部,称取6 mg Durashell C18填料,用200 μL溶液B混匀,加入上述枪头内,以1000 r/min离心5 min,制成StageTip。然后加入200 μL溶液B,以1000 r/min离心5 min洗涤StageTip柱,再加入溶液A-溶液B (1∶1, v/v)的混合液200 μL,以1000 r/min离心5 min洗涤StageTip柱,最后加入200 μL溶液A,以1000 r/min离心10 min洗涤StageTip柱。

基于StageTip柱的样品分离:将胰蛋白酶酶切后进行二甲基化修饰的样本用200 μL溶液A溶解后,加入碱性StageTip柱,以1000 r/min离心10 min,使样本充分结合在StageTip柱中的Durashell C18填料上。最后使用5%、10%、15%、20%、25%、30%和80%(v/v)的乙腈水溶液(均含0.1%TFA)各200 μL进行洗脱,将收集的滤液转干后,用ZipTip C18脱盐柱进行除盐。

### 1.8 液相色谱-质谱分析

1.8.1 乙酰化优化实验

EASY-nLC 1000高效液相色谱串联Q-Exactive用于乙酰化优化实验中的质谱检测。自制C18(粒径3 μm,孔径9 nm,美国Dikma Technologies公司)毛细管柱(100 mm×75 μm)。流动相A:2%(v/v)乙腈水溶液(含0.1%(v/v)FA);流动相B:90%(v/v)乙腈水溶液(含0.1%(v/v)FA);流速:300 nL/min。梯度洗脱程序:0~50 min, 5%B~28%B; 50~53 min, 28%B~48%B; 53~56 min, 48%B~80%B; 56~60 min, 80%B。将1.3节中除盐转干后的样本溶于10 μL流动相A中, 以15000 r/min高速离心,取4.2 μL上清进行上样分析。

Q-Exactive质谱参数为:一级分辨率为70000(*m/z* 200);一级自动增益控制(AGC)为1×10^6^;二级自动增益控制为1×10^6^;最大注射时间为60 ms;扫描范围为*m/z* 350~1300;电荷状态2~5价;碰撞归一化能量为30%;动态排除时间60 s;数据依赖采集模式为“TOP *N*”(*N*=16)。

1.8.2 蛋白质C端富集实验

EASY-nLC 1000高效液相色谱-串联Orbitrap Fusion用于蛋白质C端富集实验中的质谱检测。流速300 nL/min。梯度洗脱程序:0~13 min, 5%B~7%B; 13~33 min, 7%B~10%B; 33~88 min, 10%B~25%B; 88~110 min, 25%B~45%B; 110~113 min, 45%B~80%B; 113~120 min, 80%B。将1.7节中除盐转干后的样本溶于10 μL流动相A中,以15000 r/min高速离心,取4.2 μL上清进行上样分析。

Orbitrap Fusion质谱参数为:一级分辨率为120000(*m/z* 200);一级自动增益控制(AGC)为5×10^5^;二级自动增益控制为7×10^3^;最大注射时间为50 ms;扫描范围为*m/z* 300~1300;电荷状态1~6价;碰撞归一化能量为32%;动态排除时间60 s;数据依赖采集模式为“TOP speed”(循环时间3 s)。

### 1.9 数据分析

质谱产生的原始数据由可以调用Mascot 2.3.0搜索引擎的Proteome Discoverer 2.2按照UniProt(*homo sapiens*)数据库(版本2018.7, 95128条序列)进行搜库。

乙酰化优化实验中,统计乙酰化标记效率的搜库参数设置:酶切方式为胰蛋白酶,最大漏切位点为3个,固定修饰为Cys还原烷基化,可变修饰为Lys/Ser/Thr/Tyr乙酰化、蛋白质N端乙酰化、Met氧化。统计蛋白质C端数目的搜库参数设置如下:酶切方式ArgC,最大漏切位点为2个,固定修饰为Cys还原烷基化、Lys乙酰化,可变修饰为蛋白质N端乙酰化、Met氧化。其他参数均为一级离子质量偏差20 ppm(10^-6^),二级碎片离子的质量偏差0.02 Da。

蛋白质C端富集实验中,搜库参数设置如下:“胰蛋白酶酶切+二甲基化”策略酶切方式为ArgC, “LysargiNase酶切+二甲基化”策略酶切方式为ArgN,两者的固定修饰均为Met还原烷基化、Asp/Glu乙醇胺修饰、Lys乙酰化、肽段N端二甲基化。“胰蛋白酶酶切+乙酰化”策略酶切方式为ArgC, “LysargiNase酶切+乙酰化”策略酶切方式为ArgN,两者的固定修饰均为Met还原烷基化、Asp/Glu乙醇胺修饰、Lys乙酰化、肽段N端乙酰化。以上4种策略中,可变修饰均为蛋白质N端乙醇胺修饰、Met氧化,最大漏切位点为2个,肽段一级离子的质量偏差设为10 ppm,碎片离子的质量偏差设为0.02 Da。

数据筛选标准:肽谱匹配、肽段及蛋白水平错误发现率(FDR)<0.01,离子分数(ion score)≥20;乙酰化标记实验中,通过磷酸化修饰功能节点(ptmRS)≥0.75的标准筛选总肽谱匹配(peptide-spectrum matches, PSMs)计算标记效率。

## 2 结果与讨论

### 2.1 基于“V型”过滤装置的“一锅法”样品制备流程的建立

基于聚合物的蛋白质C端组学工作流程涉及多个化学衍生化步骤(见图1a),过量的化学试剂能够保证反应完全进行,但可能也会影响后续反应。为了避免反应之间的交叉干扰,通过对Zhang等^[18]^鉴定蛋白质C端实验流程的改进,建立了“一锅法”的富集平台(见图1b),即在还原烷基化后立即应用超滤辅助样品制备方法(FASP^[21]^)来取代猝灭步骤,从而避免过量的淬灭剂(如二硫苏糖醇或半胱氨酸等)对下一步乙酰化反应的影响。

**图 1:**
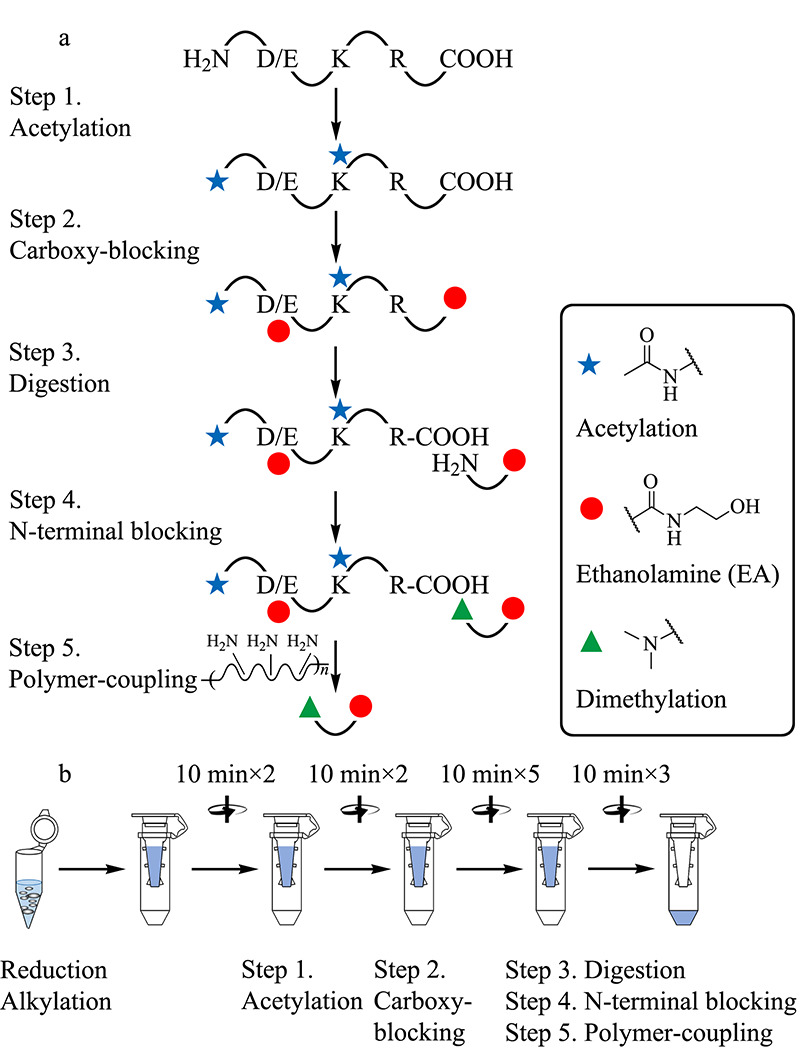
(a)基于聚合物的反向富集流程及(b)C端肽段的“一锅法”富集平台

为了提高样本的制备效率,尝试使用滤膜面积更大的“V型”过滤装置代替原本的平底过滤装置。当样本体积较大(如500 μL),使用原本的平底过滤装置,单次溶剂交换过程需要40~50 min,并且浓缩过程中产生的絮状物或沉淀容易造成滤膜堵塞。而使用“V型”过滤装置,单次溶剂交换过程仅需约10 min,从而将整个样品制备时间缩短了4~5 h。并且滤膜位于装置侧壁,样本制备过程中不易发生堵塞。对于200~400 μg的蛋白质组,两种装置可以提供相似的回收率^[21,22]^。因此,以下整个研究过程中使用基于“V型”过滤装置的“一锅法”样品制备流程。

### 2.2 乙酰化反应条件优化

蛋白质水平赖氨酸乙酰化反应是本研究重要的步骤之一,丝氨酸、苏氨酸、酪氨酸残基上的副反应会导致较高的样品复杂度和错误鉴定率。为了降低副反应的发生并提高肽段的鉴定数目,考虑从以下几个方面对乙酰化反应条件进行系统性优化:1)通过减少反应中Ac-NHS的用量以降低副反应的标记效率;2)根据以往的报道,蛋白水平乙酰化标记均是在pH 8.0的条件下进行,而碱性条件中组氨酸可能会促进相邻的羟基与Ac-NHS发生反应^[23]^,尝试调节反应的pH值,以减少副反应的干扰;3)通过在乙酰化标记后加入终浓度为500 mmol/L的NH_3_·H_2_O进行淬灭反应,在碱性较强的条件下逆转副反应的标记。基于以上思路,在进行乙酰化反应时,分别加入终浓度为10 mmol/L的Ac-NHS(pH 8.0)(条件A)、终浓度为10 mmol/L的Ac-NHS(pH 7.0)(条件B)和终浓度为5 mmol/L的Ac-NHS(pH 7.0)(条件C)。

结果表明,在条件B和条件C中,通过将反应pH调至7.0,并使用NH_3_·H_2_O进行淬灭反应,能够在保持Lys较高标记效率(>98%)(见图2a)的同时,大幅降低副反应的发生(<4%)(见图2b~2d)。由于条件C的Ac-NHS用量相对较少,因此后续实验选择条件C作为乙酰化修饰的条件。

**图 2:**
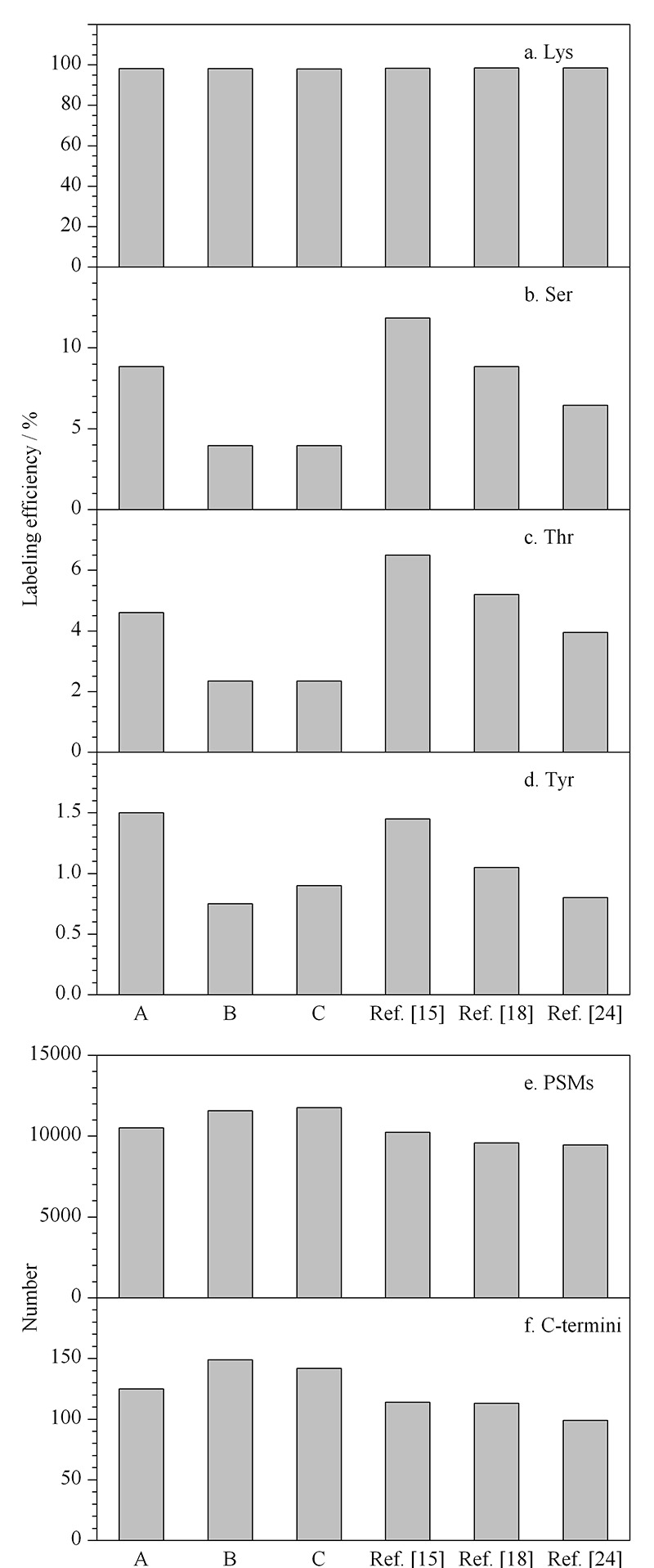
(a~d)氨基酸残基上的乙酰化标记效率以及(e)PSMs与(f)蛋白质C端的鉴定数目

此外,与以往报道^[15,18,24]^中将Ac-NHS作为乙酰化修饰试剂的方案进行了比较(见图2b~2d)。结果显示,LAACTer方法^[15]^和Zhang等^[18]^方法中乙酰化的副反应标记效率均较高,尤其是Ser的标记效率高达8%~12%。Zhou等^[24]^方法中乙酰化的副反应标记效率较低,但PSMs数目明显减少(见图2e)。并且使用条件C进行乙酰化反应比3种已报道的方案多鉴定到约15%的PSMs,比Zhou等^[24]^的方法多鉴定到约50%的蛋白质C端(见图2f)。优化后的条件能够使副反应的标记效率降到最低,且不会影响肽段的鉴定。值得注意的是,这一优化不仅有助于C端肽段的可靠鉴定,而且有助于其他Lys酰化的研究,如N端组学^[25,26]^、乙酰化位点占据率计算^[27,28]^、全局组蛋白修饰分析^[29,30]^,以及使用胰蛋白酶进行ArgC型酶切的蛋白质组学研究^[31,32]^等。

### 2.3 样品分离过程的优化

为了提高富集样本中蛋白质C端的鉴定深度,在乙酰化优化结果的基础上,以300 μg蛋白质为起始量对基于StageTip样品分离的过程进行了优化。首先用200 μL含0.1% TFA的水溶液(pH 3.0)溶解富集后的样本,加至含0.6 mg Durashell C18填料的酸性StageTip柱(以98%(v/v)乙腈水溶液A'和0.1% TFA水溶液B'代替溶液A、B制备的StageTip柱)进行蛋白质C端的鉴定实验。利用200 μL 20%(v/v)乙腈水溶液(含0.1% TFA)将肽段从StageTip柱中洗脱,在两次重复实验中,C端肽段富集效率达80%和73%,平均鉴定出133条C端肽段,对应于113个蛋白质C端(见图3a中TFA-0.6 w/o a-ion)。通过添加a离子进行搜库(该方式已被证明可以提高C端肽段的鉴定数目^[15,33]^),蛋白质C端的鉴定数目有所增加(见图3a中TFA-0.6),因此后续的数据分析中均考虑添加a离子进行搜库,但对比LAACTer^[15]^和Du等^[16]^的报道,以此方法鉴定的蛋白质C端数目仍然较少。

**图 3:**
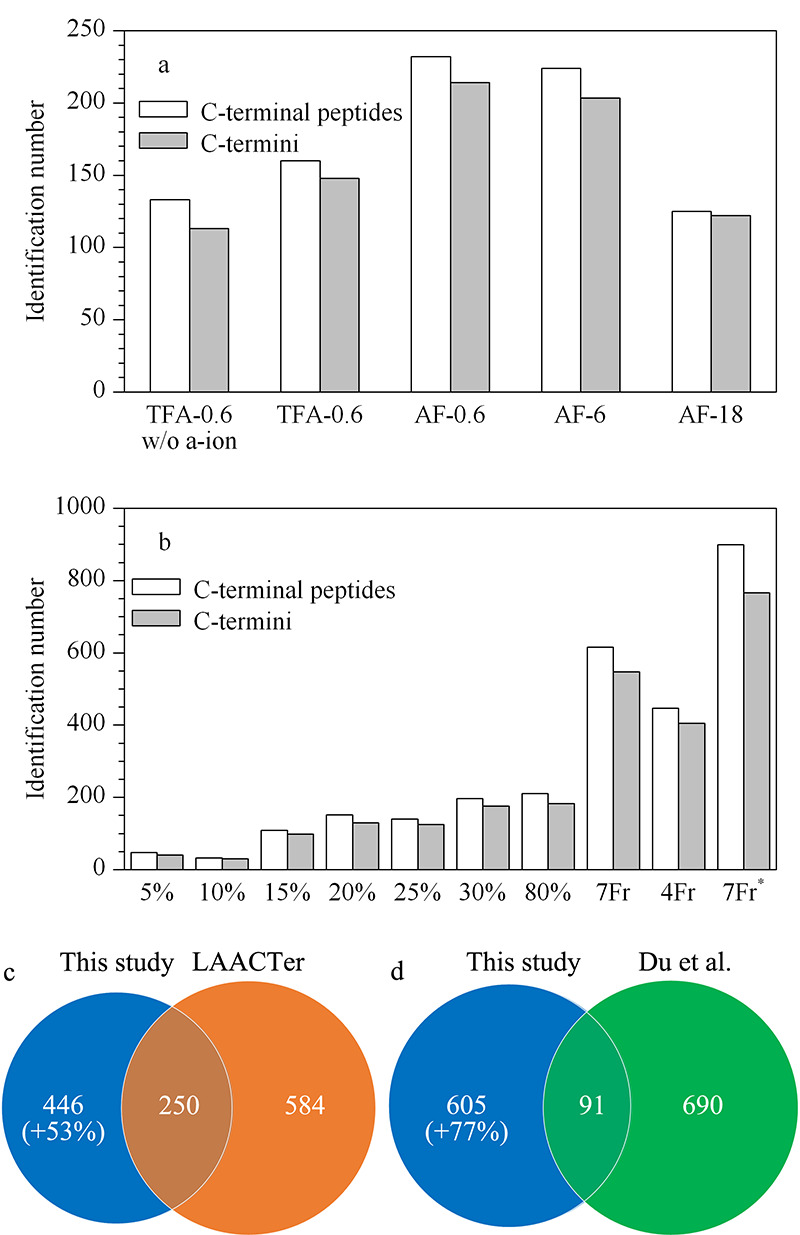
(a、b)不同条件下C端肽段和蛋白质C端的鉴定数目以及(c、d)不同策略之间的互补性分析

考虑到肽段水平反应中积累了大量有机物(包括乙酰化试剂、有机缓冲剂及其他副产物),尤其是用于肽段与聚合物偶联的EDC总量达10~20 mg,这些有机物对肽段的鉴定可能存在多方面的影响,如:1)在脱盐和nano-LC分离过程中,与Durashell C18填料竞争性结合,从而导致样本的严重损失;2)碱性EDC会对肽段信号产生压制,从而影响检测灵敏度;3)在数据依赖的采集模式中通过占据MS^2^的扫描时间,从而减少对肽段的扫描。因此,需要通过对样品分离过程进行优化以减轻这些非肽有机物造成的影响。

由于蛋白质C端的*α*-COOH被酰胺化修饰,推测在碱性环境中进行样品分离,C端肽段去质子化而极性变小,可能与Durashell C18填料有更高的亲和力,因此在洗脱的滤液中能够鉴定到更多的C端肽段。结果表明,在1.7节描述的碱性环境下进行样品分离,C端肽段的鉴定数目约比酸性环境增加了44%(见图3a中AF-0.6与TFA-0.6),因此后续基于StageTip柱的样本分离过程均在碱性环境条件下进行。

为了实现肽段和非肽类物质更好的分离,同时提升蛋白质C端的鉴定数目,进一步优化了StageTip柱的Durashell C18填料用量。结果表明,相比于使用0.6 mg Durashell C18柱填料进行样品分离,在使用6 mg柱填料时,蛋白C端的鉴定数目没有明显减少(见图3a, AF-6),且填料柱体积相对较大,更利于进行将样本洗脱为多个组分的实验操作;而使用18 mg柱填料时,大量填料可能由于非特异性吸附作用引起样品损失,蛋白质C端的鉴定数目约下降了43%(见图3a, AF-18),不利于后续进一步的优化实验。

因此,通过使用含6 mg Durashell C18填料的StageTip柱,将样本依次用5%、10%、15%、20%、25%、30%和80%(v/v)乙腈水溶液(均含0.1% TFA)各200 μL洗脱为7个组分,约93%的蛋白质C端仅在一个或两个组分中鉴定到,表明将样本洗脱为多个组分后,降低了样品的复杂度,达到了比较好的分离效果,更加利于蛋白质C端的鉴定。两次重复实验中平均鉴定到616条C端肽段,对应547个蛋白质C端(见图3b中7Fr),总数目约为酸性条件下的4倍。在两次重复实验中,约73%的蛋白质C端被鉴定到两次,皮尔森相关系数达0.91,表明实验的重复性较好。考虑到蛋白质C端数目约占总PSMs的1%(见图2e和2f),以300 μg的蛋白质组为起始量进行样品分离过程的优化实验,估计C端肽段仅约为1~3 μg,但将洗脱后的7个组分组合成4个组分(5%、10%+25%、15%+30%、20%+80%)后进行质谱分析,鉴定数目约下降了30%(见图3b中4Fr),证明了富集后样本的仍具有较高的复杂性及需要更高分离度的必要性。

综上所述,通过将300 μg蛋白质组富集的样品洗脱为7个组分,在两次重复实验中共鉴定出732条C端肽段,对应696个蛋白质C端,达到本课题组前期LAACTer方法^[15]^和Du等^[16]^的鉴定深度(分别鉴定到834和781个蛋白质C端)。

与LAACTer策略^[15]^(切割Lys/Arg残基N端,产生以Lys或Arg开头且*α*-N端带有乙酰化修饰的C端肽段)的研究结果相比,本研究的策略(切割Arg残基C端,产生*α*-N端带有二甲基化修饰的C端肽段)鉴定到的蛋白质C端种类新增约53%,且重叠部分低于20%,两者总数目达1200以上(见图3c);与Du等^[16]^的策略(切割Lys残基C端,产生*α*-N端带有二甲基化修饰的C端肽段)相比,本研究鉴定到的蛋白质C端新增约77%,重叠部分低于7%,两者总数目达1380以上(见图3d)。与Du等^[16]^的研究(先将蛋白质组进行样品分离,再对每个组分进行富集)相比,本研究中的策略是先进行目的肽段富集再完成样品分离,避免了样品损失,大大简化了操作流程。不同策略中使用不同的蛋白质水平衍生化修饰、酶切方式以及肽段N端衍生化方式,产生了含不同*α*-N末端结构的C段肽段,因此理论上不同策略对蛋白质C端的鉴定有着各自独特的偏好性,两两之间的鉴定结果有所不同,这不仅实现了对蛋白质C端的深度鉴定,而且在鉴定范围上具有独特性,将为蛋白质C端机制的深入研究提供新的有力工具。

值得注意的是,696个蛋白质C端是经过严格的数据筛选得到,即1)肽段水平FDR<0.01; 2)ion score≥20; 3)C端带有乙醇胺修饰。若按Du等^[16]^所报道的FDR<0.01的置信标准,两个重复实验中平均鉴定到766个蛋白质C端,总数目可达933个(见图3b, 7Fr^*^),这是基于聚合物富集C端肽段策略获得的最大数据集之一^[15,16,17,18,19,20]^。

### 2.4 利用新型ArgN型酶切与肽段N端衍生化进一步扩大蛋白质C端鉴定范围

LysargiNase是一种可以特异性切割Arg和Lys残基N端的胰蛋白酶镜像酶^[34]^,可以在酶切所得的肽段N端留下两种碱性氨基酸。LysargiNase与胰蛋白酶在全蛋白质组肽段的鉴定覆盖范围具有一定的互补性^[34,35]^,但二者在鉴定Arg特异性酶切所产生的C端肽段上的互补性鲜有研究。同时,考虑到肽段N端的化学保护基会影响肽段的色谱保留和质谱检测,推测C端肽段N端上不同的衍生化修饰在进行蛋白质C端鉴定时可能会存在一定的互补性。

因此,为了进一步扩大蛋白质C端鉴定范围,本研究探索了两种酶(LysargiNase和胰蛋白酶)和两种肽段N端衍生化修饰的组合对C端肽段鉴定数目和种类的影响。在两次重复实验中,通过对未分级分离的C端肽段样品进行单针质谱分析,即仅使用200 μL 20%(v/v)乙腈水溶液(含0.1% TFA)将富集后的样品从StageTip柱洗脱。在两次重复实验中,4种策略共鉴定到372个蛋白质C端。

其中“胰蛋白酶+二甲基化”策略鉴定到的蛋白质C端数目共计220个(见图4a和4b)。新型“LysargiNase+乙酰化”策略在前者策略的基础上,鉴定到的蛋白质C端种类新增105个(见图4b),两种策略鉴定的蛋白质C端数目占总数目的87%,且两种策略鉴定蛋白质C端种类的重叠部分仅为20%(见图4b),说明两种策略对于蛋白质C端的鉴定范围具有很强的互补性。

**图 4:**
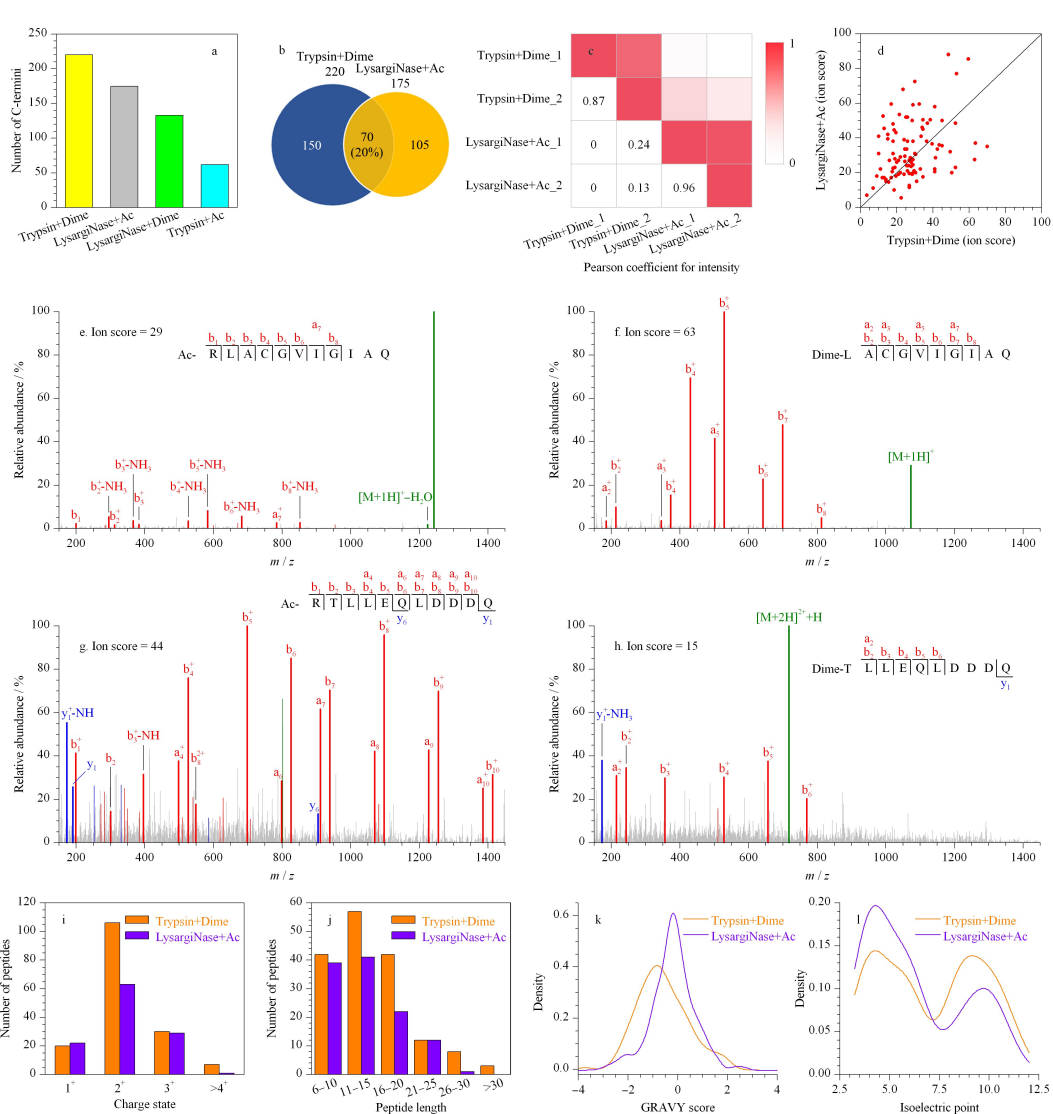
不同蛋白酶和N端衍生化组合对鉴定蛋白质C端的互补性分析

对两种策略下鉴定到的C端肽段进行对比分析,发现共有肽段的强度相关性较差(见图4c),且离子分数差异较大(见图4d),表明两种方法对序列相同的C端肽段具有不同的偏好性。这种偏好性可归因于两种方法产生了不同的N末端结构:乙酰化的*α*-N端(由“LysargiNase+乙酰化”策略产生)和二甲基化的*α*-N端(由“胰蛋白酶+二甲基化”策略产生)。一方面,“LysargiNase+乙酰化”策略中,Arg上胍基强碱性的优势在于较高的离子化效率利于肽段的检测^[36]^;但该基团的局限可能在于碱性太强而更难碎裂^[37,38]^,从而影响C端肽段的鉴定(见图4e和4f)。另一方面,“LysargiNase+乙酰化”策略中*α*-N端上的乙酰化修饰通过增强肽段主链解离和稳定N端碎片离子^[39,40]^,能产生更多的a离子和b离子(见图4g和4h)。此外,乙酰化的*α*-N端Arg基团的疏水性远低于二甲基化的*α*-N端(XLOGP3^[41]^值分别为-2.84和-0.2),这在很大程度上影响了肽段的色谱保留,从而影响了对蛋白质C端的鉴定。

为了深入了解两种策略对鉴定蛋白质C端的偏好性,分析了两种策略各自“特有的”C端肽段的特征。结果显示,“胰蛋白酶+二甲基化”策略更倾向于鉴定长度为11~15的2^+^肽段(见图4i和4j)。而“LysargiNase+乙酰化”策略更倾向于鉴定长度为6~15的2^+^肽段,以及疏水性较低且含有较多酸性残基的肽段(见图4k和4l)。

综上所述,通过使用基于Arg型酶切的新策略“LysargiNase+乙酰化”,在原有策略“胰蛋白酶+二甲基化”的基础上进一步扩大了蛋白质C端的覆盖范围。

## 3 结论

本研究通过对基于Arg型酶切的反向富集策略的系统性优化,缩短了样本准备时间,提升了C端肽段的鉴定深度,并扩大了C端肽段的鉴定范围。此外,通过与其他鉴定C端肽段的策略相比,本研究在鉴定范围上具有独特性,进一步提示,可以采用多种策略联用的方式进行蛋白质C端的鉴定,用以提升蛋白质C端的鉴定“深度”和“广度”。通过将优化后的方法与其他策略结合,不仅可以高效地用于基于Arg型酶切的蛋白质C端组学深度分析,还可以对不同细胞、组织来源的蛋白质C端状态进行系统表征,获得蛋白质C端状态的全景,将为蛋白质C端在生理病理中的分子机制研究提供新的信息。
